# Peer‐led interventions to prevent tobacco, alcohol and/or drug use among young people aged 11–21 years: a systematic review and meta‐analysis

**DOI:** 10.1111/add.13224

**Published:** 2016-02-09

**Authors:** MacArthur Georgie J., Harrison Sean, Caldwell Deborah M., Hickman Matthew, Campbell Rona

**Affiliations:** ^1^ School of Social and Community MedicineUniversity of BristolBristolUK

**Keywords:** Alcohol, cannabis, intervention, peer, substance use, systematic review, tobacco, young people

## Abstract

**Background and Aims:**

Peer‐led interventions may offer a beneficial approach in preventing substance use, but their impact has not yet been quantified. We conducted a systematic review to investigate and quantify the effect of peer‐led interventions that sought to prevent tobacco, alcohol and/or drug use among young people aged 11–21 years.

**Methods:**

Medline, EMBASE, PsycINFO, CINAHL, ERIC and the Cochrane Library were searched from inception to July 2015 without language restriction. We included randomized controlled trials only. Screening and data extraction were conducted in duplicate and data from eligible studies were pooled in a random effects meta‐analysis.

**Results:**

We identified 17 eligible studies, approximately half of which were school‐based studies targeting tobacco use among adolescents. Ten studies targeting tobacco use could be pooled, representing 13 706 young people in 220 schools. Meta‐analysis demonstrated that the odds of smoking were lower among those receiving the peer‐led intervention compared with control [odds ratio (OR) = 0.78, 95% confidence interval (CI) = 0.62–0.99, *P* = 0.040]. There was evidence of heterogeneity (*I* ^2^ = 41%, χ^2^ 15.17, *P* = 0.086). Pooling of six studies representing 1699 individuals in 66 schools demonstrated that peer‐led interventions were also associated with benefit in relation to alcohol use (OR = 0.80, 95% CI = 0.65–0.99, *P* = 0.036), while three studies (*n* = 976 students in 38 schools) suggested an association with lower odds of cannabis use (OR = 0.70, 0.50–0.97, *P* = 0.034). No studies were found that targeted other illicit drug use.

**Conclusions:**

Peer interventions may be effective in preventing tobacco, alcohol and possibly cannabis use among adolescents, although the evidence base is limited overall, and is characterized mainly by small studies of low quality.

## Introduction

Engagement in risk behaviours such as tobacco, alcohol, cannabis and other illicit drug use has multiple negative health consequences, including respiratory problems, violence, injury, sexual risk behaviour, poorer educational attainment, psychosis, mental illness, risk of dependence, morbidity and mortality later in life 1, 2, 3, 4, 5, 6, 7. While the rate of substance use among young people in the United Kingdom is decreasing overall, a substantial proportion of young people continue to use these substances, with 31% of 16–24 year olds in the United Kingdom having ever used cannabis and nearly one‐fifth being regular or occasional smokers 8, 9. Critically, alcohol use among young people in the United Kingdom is particularly high compared to other European countries, with one‐third of young people aged 15–16 reporting hazardous drinking 8, 9.

Evidence regarding the effectiveness of programmes to prevent substance use among young people is mixed, and there remain gaps in the evidence base 10, 11, 12, 13, 14, 15, suggesting that there is substantial scope for the development of novel approaches that target tobacco, alcohol or cannabis use during adolescence. Peer‐based approaches may offer a potentially effective approach for addressing substance use, but such interventions have received comparatively less attention in relation to substance use in recent years compared to the 1980s and 1990s.

Peer education has been defined as ‘the teaching or sharing of health information, values and behaviours between individuals with shared characteristics’ 16. Such an approach may involve the delivery of part or all of an intervention by same age or older peers in informal or formal settings, such as community centres, street settings, nightclubs, school classrooms or youth clubs, using pedagogical or ‘diffusional’ methods (i.e. where peer‐led education occurs as part of the normal communication within social groups) 17, 18. The promise of such approaches is borne of a notion that young people learn from each other, that peers have greater credibility among young people, have a shared cultural background and that they may have a greater understanding and empathy surrounding the health behaviour of young people. They may also act as positive role models who can reinforce behavioural messages 17.

To date, peer‐based programmes have been employed to target substance use, sexual risk behaviour, HIV prevention and psychosocial wellbeing among young people 19, 20, 21, 22, 23, 24, 25, and there is promising evidence from existing intervention models 19, 22, 26. Earlier systematic reviews suggested that there was evidence that peer interventions could change behaviour, as well as improve knowledge 18, 27, 28. Notably, process evaluations showed that young people reported positive views towards peer interventions, such as: finding peer‐led sessions fun, feeling that peers are credible sources of information and preferring peer‐led sessions over teacher‐led sessions 27.

To date, no systematic reviews have focused solely on the impact of such interventions on substance use behaviour, and no systematic review has been conducted in the field in the past decade. As such, it is not known whether such interventions could be beneficial in preventing substance use among young people, whether they could prevent single or multiple behaviours and whether any effect could be quantified. We conducted a systematic review and meta‐analysis to assess and quantify the impact of peer‐led intervention models on tobacco, alcohol and illicit drug use among young people (here defined as those aged 11–21 years). The review was conducted with a view to identifying particular intervention models or components that could be used as the basis for new programmes to prevent harm from substance use among young people in the United Kingdom. We hypothesized that peer‐led interventions would be effective in preventing the use of these substances.

## Methods

The protocol for this systematic review is registered with PROSPERO (registration number CRD42014009790) 28. The primary objective was to identify and review the effects of peer‐led interventions that aim to prevent tobacco, alcohol and/or illicit drug use among young people aged 11–21 years. This age group was chosen as it encompasses young people in secondary and tertiary education, at which stage substance use may be initiated and sustained, and at which point preventive interventions may be targeted. While we aimed to review interventions targeted to any illicit drug, all studies focused on cannabis use, thus we refer to this particular drug in this paper. To be classed as a peer‐led intervention, programmes needed to include a substantial component in which peers were involved in the delivery of the intervention; for instance, via the direct delivery of curriculum components, or by acting as a mentor or ‘buddy’ to study participants.

### Search strategies

Literature searches of databases were conducted without language or geographical restriction from inception to July 2015 in Medline, Embase, PsycINFO (all via OvidSP), CINAHL (via EBSCOhost), ERIC (via ProQuest), the Australian Education Index, British Education Index and the Cochrane Library (via Wiley Online Library). Based on the authors’ own knowledge and advice from colleagues in the field we searched for the most relevant grey literature by prioritizing and checking websites of organizations renowned in the field, such as the Joseph Rowntree Foundation, World Health Organization, Evidence for Policy and Practice Information and Co‐ordinating Centre (Eppi Centre) and National Youth Agency. Search terms included MESH and text word combinations relating to young people, the behaviours of interest, peer‐leaders and randomized controlled trials (RCTs). The Medline search strategy is provided in Supporting information, Table S1 and via the PROSPERO record 28. Searches in other databases were adapted accordingly.

Titles and abstracts of the studies identified were screened by two independent researchers (G.J.M., S.H.) and disagreements were resolved by discussion. Two reviewers (G.J.M., S.H.) screened full texts of identified studies to determine whether they met criteria for inclusion. Disagreements were resolved by discussion or by a third reviewer where necessary (R.C., M.H.). Two studies identified in languages other than English (Norwegian and German) were assessed for inclusion and, if included, were translated in full by an individual fluent in that language.

Studies were eligible if they targeted tobacco, alcohol and/or drug use and included young people aged 11–21, or if more than half the participants were aged within this bracket. Comparators could be usual practice, no intervention or teacher, adult or professional‐led intervention. Exclusion criteria included the following: interventions targeting young people with a clinical disorder; those that targeted young people and another population group such as parents; multi‐component interventions; brief interventions, clinical interventions; studies with less than 6 weeks of follow‐up; studies that targeted prescription drug or body‐enhancing drugs; and study designs other than RCTs, including pilot RCTs and feasibility studies. There was no limit on the setting of the intervention.

## Study outcomes

Outcomes that could be included were those relating to tobacco use (including smokeless tobacco) or alcohol use, such as frequency of use or volume consumed. Those relating to the use of drugs including cocaine, ecstasy (3,4‐methylenedioxy‐methamphetamine: MDMA), glue, gas, aerosol, solvents, magic mushrooms, crack, ketamine, heroin, poppers, lysergic acid diethylamide (LSD), methamphetamine and amphetamine could also be included.

## Data extraction and analysis

Data were extracted in duplicate by G.M. and S.H. working independently using piloted forms, including details relating to the intervention (date, setting, duration, country, behaviours targeted), baseline characteristics of participants, duration of follow‐up, theoretical underpinning, outcome measures and effect sizes. Methods of analysis of data required for meta‐analyses were checked by another researcher (D.C.). Outcome data were extracted for the most harmful measure of the target behaviour(s), e.g. weekly rather than monthly smoking where numbers were sufficient. Data were extracted from the end of the intervention or the next closest time point.

In the first instance, our preference was to extract the unadjusted odds ratio and 95% confidence intervals (CI) directly from papers. Where this was not reported, odds ratios (ORs) were calculated in Stata (version 13.1) using the raw data 30, 31, 32, 33, 34, 35, 36. For continuous outcomes, the standardized mean difference (SMD) was calculated and converted to a log OR 20, 37, 38, 39 using standard methods 40 (InlogOR = d(π/sqrt)^3^). To overcome unit of analysis error where data were presented separately by gender 35, 37, or where there were multiple experimental arms 39, groups were merged to enable synthesis [Ref 41, section 7.7.3.8]. Where pre‐ and post‐test continuous data were presented and the data between groups were markedly different at baseline 38, 39, the mean change and standard deviation (SD) for change from baseline were calculated, as recommended 41, 42. For a minority of studies additional assumptions were made to enable inclusion in meta‐analyses (see Supporting information, Material S2); for example, adjusting for clustering where this had not been conducted 41. For the latter adjustments, the intracluster correlation coefficient (ICC) reported by Campbell *et al*. 19 was used (see Supporting information, Material S2). These assumptions were examined in sensitivity analyses.

Risk of bias was assessed using the Cochrane tool 41. Publication bias was assessed with a funnel plot and by using the Egger test, which quantifies the bias shown in the funnel plot by regressing the standard normal deviate on precision 43.

## Synthesis of results

Separate syntheses were conducted for studies that targeted tobacco, alcohol or cannabis use and those that included adjusted or unadjusted effect estimates, as some studies adjusted for baseline differences. Several studies did not provide baseline data to facilitate such adjustments across all studies. Heterogeneity was assessed with the *I* ^2^ statistic and χ^2^ test. We anticipated heterogeneity and planned to use the random effects model for synthesis; thus, unless indicated otherwise, all results presented here are from the random effects model. However, a fixed effect model was conducted as a sensitivity analysis. We did not identify sufficient studies to conduct the pre‐planned subgroup analyses or to compare peer‐led interventions to teacher‐ or professional‐led interventions; instead, the latter studies were included in sensitivity analyses.

## Results

**Figure 1:**
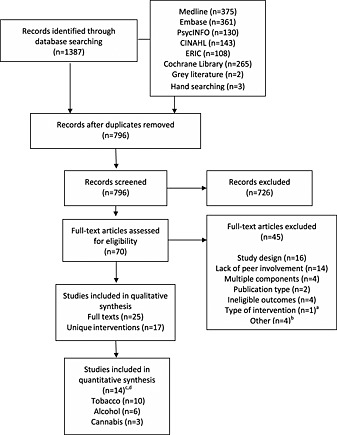
Flow of studies in systematic review. ^a^Brief intervention; ^b^ British Library were unable to obtain two papers; one study failed to report control data; one study targeted individuals aged more than 21 years. ^c^Three of the 17 studies could not be included in quantitative syntheses. ^d^Three of the 15 studies targeted all three substances. (Flowchart template obtained from Moher D, Liberati A, Tetzlaff J, Altman DG, The PRISMA Group (2009). *P*referred *R*eporting *I*tems for *S*ystematic Reviews and *M*eta‐*A*nalyses: The PRISMA Statement. PLoS Med) 6(6): e1000097)

Figure 1 shows the total number of studies identified, screened and reviewed and the reasons for exclusion. A total of 796 unique studies were retrieved, and from these, 25 eligible papers were identified, representing 17 unique studies (eight were papers reporting on the duplicate studies) 44, 45, 46, 47, 48, 49, 50, 51. Ten of these unique studies could be included in the quantitative synthesis relating to tobacco smoking; six could be included in quantitative syntheses relating to alcohol; and three were synthesized in relation to cannabis use.

One study 52 was excluded from the main meta‐analysis on the basis that only data relating to daily smoking among baseline smokers were reported. In this study, the odds of smoking cessation were higher in the peer intervention arm compared to the control arm (OR for smoking cessation = 3.73, 95% CI = 1.00–13.89, *P* = 0.01), but the effect was not sustained at 1 year (OR = 0.60, 0.11–3.31, *P* = 0.440). Another study 53 was excluded as it reported data relating to substance use only as a composite variable. This study demonstrated that frequency of attendance at peer mentoring sessions was associated with lower risk of substance use among adolescents aged 12–15 who had a parent with HIV (regression coefficient − 0.18, *P* = 0.028). Lastly, a third study was excluded as insufficient data were provided from the study sample.

Table 1 summarizes the characteristics of the included studies. Approximately half of the studies (n=9 of 17, 52%) targeted tobacco smoking and most were conducted in the United States. The remaining studies were conducted in the United Kingdom 19, Australia 35, Norway 39, Spain 36, Poland and Romania 54, while a multi‐centre study was conducted in Australia, Norway, Chile and Swaziland 51. A number of studies targeted young people aged 12–13 years (*n* = 8), while four studies targeted young people aged 13–18 and most were conducted in the school setting. Sample sizes (adjusted for clustering by the authors where necessary) varied between *n* = 100 and 9811 for tobacco use; *n* = 100 and 363 for alcohol use; and *n* = 101 and 516 for cannabis use, with the number of schools randomized to each arm varying between two and 30. Several school‐based interventions used a similar theoretical model, the social influences model, to underpin the curriculum, with the intervention addressing factors such as managing pressure to use substances, awareness of the impacts of advertising and practice of resistance skills (Table 1).

**Table 1:** Characteristics of included studies.

*Author*	*Name, location*	*Sample size (n)*	*Age (average) (years)*	*Behaviour*	*Setting*	*Intervention*	*Follow‐up*	*Theory*	*Findings*
Albrecht 2006, 1998^a^	Teen Fresh Start PA, USA	142	14–19 (17)	Tobacco	Unclear	8‐week group intervention. Didactic content about smoking and pregnancy Buddy to provide social support and to reinforce smoking cessation strategies	1 year	Cognitive behavioural theory incorporating Jessor's problem behaviour theory	Lower odds of smoking in intervention arm involving peers compared to control at 8 weeks follow‐up (OR = 0.27, 95% CI = 0.10–0.73), but no difference between groups evident at 1 year (OR = 1.67, 95% CI = 0.47–5.99)
Armstrong 1990; Shean 1994	— Australia (Western)	2366	12–13 (12)	Tobacco	Primary school	5 sessions over 6 months. Information on negative consequences and physiological effects; addressing peer norms; practice of refusal techniques; discussion of arguments for and against smoking; considering role of family; analysis of advertising; public commitment not to smoke	1, 2 and 7 years	Social consequences curriculum	Girls: fewer girls in the intervention group commenced smoking compared to control (23.2 versus 33.1%) at 1 and 2 years (37.8 versus 49.7%). Adjusted difference in prevalence at 1 year: –7.8%, −17.1 to 1.5% Boys: a higher proportion of boys in the peer‐led arm smoked compared to control at 1 year (34.7 versus 29.4%) and 2 years (41.9 versus 33.5%). Adjusted prevalence 4.9%, −4.7 to 14.5%. OR for any smoking in past 12 months (boys and girls) = 0.87, 0.61–1.23^b^
Bobrowski 2014	Based on components of Project Northland Poland (Warsaw)	802	10–11	Alcohol	Primary school	1. 5 sessions plus activities to be completed at home. Sessions covered: underage drinking, advertising, consequences of drinking, and peer pressure through booklets containing cartoons 2. 6 group sessions involving role play, games and activities around: peer pressure, values and goals, reasons for drinking, consequences, resistance and alcohol‐free leisure time	15 and 27 months	Adapted from a program based on theory of reasoned action, problem behaviour theory and social learning theory	At 27 months among younger students, no difference in alcohol use rate between groups (*F* = 1.21, *P* = 0.307), but data suggested an effect on delay of initiation of drunkenness among younger and older students (χ^2^ = 4.69, *P* = 0.030).
Botvin 1990, 1984	Life skills training NY, USA	1311	12–14	Tobacco Alcohol Cannabis	Junior high school	20 sessions +10 session booster the following year Consequences of substance use; decision‐making; resisting social influences e.g. friends and advertising; coping with anxiety, interpersonal and communication skills; assertiveness Booster reinforced messages from the intervention	1 year	Cognitive behavioural approach	Lower proportion of daily, weekly and monthly smokers in peer‐booster condition compared to control (5 versus 16%; 3 versus 13%, 40 versus 74%, respectively). OR = 0.33, 0.08–1.32. No marked difference between study arms for weekly or monthly alcohol use, or for frequency or drunkenness; although participants in peer‐booster arm reported consuming less alcohol per occasion compared to control. OR = 0.88, 0.32–2.39. Lower monthly marijuana use in peer booster arm compared to control (5 versus 13%) but no difference for daily, weekly or ever used^c^ OR = 0.24, 0.03–2.23
Campbell 2008	ASSIST England and Wales	10 730	12–13	Tobacco	School	10‐week period of informal conversations between peer supporters and pupils in year group	1 year 2 years	Diffusion of Innovation	Lower odds of smoking in the past week among all students in the intervention arm compared to control immediately after intervention (aOR = 0.75, 0.55–1.01, *P* = 0.06), at 1 year (aOR 0.77, 0.59–0.99, *P* = 0.04) and 2 years (aOR 0.85, 0.72–1.01, *P* = 0.07). Overall effect across all three follow‐ups: OR = 0.78, 0.64–0.96. No additional benefit for occasional, experimental or ex‐smokers at baseline
Elder 1993; Eckhardt 1997	— CA, USA	2668	11–16 (12)	Tobacco	School	7th grade: 6 weekly lessons in autumn and 4 monthly lessons in spring covering: video, discussion and role play around health and social consequences, antecedents, resisting peer pressure, decision making, addiction and cessation and performance of skits re refusal. 8th grade: 8 monthly lessons covering refusal skills, protest to advertising, community action projects, communication skills. 9th grade: direct mail and telephone calls to deliver preventive messages or cessation advice	End of intervention (3 years)	NS	Prevalence of tobacco use [smokeless tobacco (ST) and smoking] lower in intervention arm compared to control (14.2 versus 22.5%) at final follow up (end of intervention) largely due to difference in smoking rather than ST use. OR for combined tobacco use = 0.57, 0.36–0.88^b^
Ellickson & Bell 1990, 1990b	Project ALERT CA and OR, USA	6527	12–14	Tobacco Alcohol Cannabis	School	7th grade: 8 participatory weekly lessons involving role play, group exercises and skills practice covering: reasons not to use substances, identification of pressures to use substances, saying no to pressure, countering pro‐drug messages and the benefits of resistance. 8th grade: 3 booster lessons to reinforce the 7th grade programme	End of intervention (15 months)	Builds on the social influence model and draws on the health belief model and self‐efficacy theory of behaviour change	Tobacco: little substantial difference in the rate of smoking in the past month among baseline non‐users in intervention compared to control arm (7.1 versus 8.4%). Lower rate of daily, weekly and monthly smoking among experimental users in intervention group compared to control (2.3 versus 5.1%; 5.7 versus 11.1%; 16.5 versus 22.4%) at end of intervention (15 months). OR (non‐users) = 1.20, 0.60–2.41; OR (experimental users) = 0.49, 0.23–1.06. Alcohol: no marked difference in past month alcohol use among baseline non‐users; or for weekly or monthly alcohol use among experimental users at 15 months. OR (non‐users) = 1.12, 0.66–1.91. Cannabis: lower initiation rate among non‐users in intervention arm compared to control (8.3 versus 12.1%). Little difference between groups for monthly use. OR (non‐users) = 0.79, 0.40–1.56^b^
Fromme & Corbin 2004	Life‐style management class (LMC) TX, USA	576	18–19	Alcohol	University	Two 2‐hour group meetings to: increase knowledge about drinking patterns and consequences; peer norm correction; increase motivation to reduce heavy alcohol use; provide skills in the management of alcohol use and stress	6 months	NS	Decrease in alcohol use from pre‐test to post‐test in both study arms and no marked difference between groups. OR = 0.98, 0.63–1.51^b^
Lotrean 2010	Romania	1196	13–14 (13.7)	Tobacco	School	5 weekly sessions of 45 minutes including videos of interviews with young people and real‐life situations, and small group activities led by peers	9 months	Social cognitive theory, integrated model of change, social influences approach	Fewer regular smokers in intervention arm compared to control at follow‐up (4.5 versus 9.5%). OR for regular smoking onset = 0.45, 0.19–1.04^b^
Luna‐Adame 2013	Life skills training Spain	1048	10–14 (11)	Tobacco	Secondary School	24 1‐hour group sessions in the first year and 12 sessions in the second year covering: social skills, problem solving, correction of norms and enhancement of self‐esteem. ‘Good behaviour game’ used in programme sessions in socio‐economically disadvantaged neighbourhoods	End of interventionand 1 year	NS	No difference in overall tobacco consumption rate between intervention and control groups at end of intervention and 1 year follow‐up (Mann–Whitney *U*‐test = 119 640.0, *P* > 0.05; and 10 163.5, *P* > 0.05, respectively)
Murray 1988, 1987^d^	— MN, USA	3820	12–13 (12)	Tobacco	Junior high school	Curriculum with 5 sessions over 6 months regarding: social forces that encourage smoking; negative consequences; peer norm correction; skills to resist pressure; management of family smoking; exercises around advertisements	1, 2, 3, 4 and 5 years	Social influences model	Lower incidence of ever smoking among baseline non‐smokers in the peer‐led arm compared to the control (adult‐led arm) at 2 years (5.7% difference), but not at 1 year. Lower incidence of daily and weekly smoking among control arm at 1 (2.3 and 2.1% difference), and 2 years (2.6 and 1.7% difference, respectively). OR for weekly smoking = 1.18, 0.23–6.13^b^
Perry 1989; Perry & Grant 1991	WHO collaborative study on alcohol and young people Australia, Chile, Norway, Swaziland	2536	11–18^e^ Australia (13.1) Chile (14.2) Norway (14.3) Swaziland (15.6)	Alcohol	School	4 × 50‐minute weekly whole class or group discussion sessions plus a booster and review 1 month after the fourth session. Sessions covered: the social and health consequences of alcohol use; prevalence; changing normative expectations; reasons for non‐use; alternatives to drinking; peer pressure; practice of refusal; advertising; and dealing with alcohol use in social situations. The programme was tailored to each country	3 months	Social learning theory	Lower alcohol‐use score in peer‐led programme (3.15 ± 0.12) compared to teacher‐led (3.46 ± 0.12) or control arms (3.52 ± 0.16) for non‐drinkers (scores pooled for all countries although same general pattern noted across countries). OR = 0.67, 0.46–0.98^b^
Rosenblum 2005	Peer mentoring NY, USA	157	9–15 (11.4)	Tobacco Alcohol Cannabis	Community	Attendance at support groups; computer use; field trips; instruction in arts, craft, poetry, public speaking; coping with peer pressure; educational films; tutoring	1 year	–	Attendance at peer mentoring sessions associated with lower substance use risk (β −0.18, *P* = 0.028)
Severson 1991	Project PATH OR, USA	2,552^f^	12–14	Tobacco	School	7‐session programme over 2–3 weeks involving use of video targeted to highlighting pressures to use tobacco and on effective ways to respond to pressures. Content covered: decision‐making; consequences of using tobacco; addiction; advertising; prevalence; public commitments and interviews with adults about their tobacco use	1 year	Social influence model	Smokeless tobacco (ST): no treatment effect for baseline non‐users; but higher cessation rate among baseline chewers (OR = 10.45, 1.93–56.64). Smoking tobacco: subjects in both study arms increased smoking by similar amounts. OR = 1.13, 0.75–1.69^b^
Telch 1990	— TX, USA	572	12–13	Tobacco	School	5 sessions over 3 weeks. Interactive videotape program regarding negative consequences; peer, social and media pressures; resistance strategies; refusal techniques; advertisements; and resisting media appeals	6 months	Social influences approach	Onset of experimental and regular smoking among baseline non‐users lower in peer‐led arm compared to control (2.1 versus 8.0%; 0.0 versus 2.5% respectively). For all participants, lower rate of regular smoking in peer‐led arm compared to control (0.8 versus 5.5%), no marked difference for experimental smoking^g^ OR for regular smoking = 0.26, 0.03–2.24^b^
Valente 2007	TND Network CA, USA	541	16.3 (average)	Tobacco Alcohol Cannabis Cocaine	School	12 sessions over 3–4 weeks with a focus on skills, motivation and decision making. Information was provided around health and social consequences; correction of misperceptions; stress management; coping skills; communication skills; and tobacco cessation	1 year	NS	Receipt of TND network (i.e. peer‐led arm) was not associated with decreased tobacco (b = −0.40, −1.19 to 0.40) or alcohol use (b = −0.43, −1.63 to 0.77), but was associated with decreased marijuana (b = −0.64, −1.09 to –0.19), cocaine use (b = −0.37, −0.63 to –0.10) and composite substance use (b = −0.37, −0.54 to –0.20). There was a higher likelihood of quitting substance use in the peer‐led arm (OR = 3.41, 1.68–6.92, *P* < 0.01)
Wilhelmsen 1994	— Norway, Bergen	955	12–13	Alcohol	School	10 sessions over 2 months regarding traditions in using alcohol; norms; management of pressure; and attitudes towards alcohol use. Participants also interviewed friends and family regarding social norms	2–4 weeks	Social cognitive theory	Alcohol use was lower in the peer‐led arm (highly‐role specified peer arm) compared to control [mean alcohol use 0.53 (SD 1.0) versus 0.69 (SD = 1.3)] but higher among those in the less role specified peer arm compared to control [mean 0.90 (SD = 1.4) versus 0.69 (SD = 1.3)]

a More than one study is listed where there were duplicate articles from the same intervention study. The primary study for such interventions is listed first.

b Odds ratios (OR) calculated from data presented in papers, adjusted for clustering where appropriate. Confidence intervals (CIs) are 95% CIs.

c Data are presented for peer‐led arm plus booster and control, as per pre‐agreed data extraction plan.

d Findings were extracted from study II only, as this study was deemed to better reflect real world conditions. Study arms compared were peer‐led social influences programme with videotape supplements and adult‐led social influences programme with videotape supplements.

e This intervention targeted young people aged 13–14 years, but included young people of a wider age range owing to the multi‐country nature of the study.

1434 of these individuals in middle‐school received the peer‐led intervention.

f Comparisons reported are for peer‐led arm and control group 1 (as only this control group was included at the time of randomization). NS = not stated; OR = odds ratio; aOR = adjusted odds ratio; TND = towards no drug abuse; ASSIST: a stop smoking in schools trial; PATH: programmes to advance teen health.

As expected, the included studies were heterogeneous overall. The duration of interventions was highly variable, ranging from a matter of weeks to more than 2 years (equivalent to 3 school years) (see Supporting information, Table S3), although the school‐based nature of interventions meant that the duration was often extended over a matter of years despite there being relatively few taught classes in each school year. The number of sessions in the peer‐led programmes ranged from two to 36, but for most studies (*n* = 11) the number ranged between five and 12 sessions (see Supporting information, Table S3). There was also variation between studies in the follow‐up period, which ranged from the end of intervention 30, 31, 52 to 7 years 50, although the maximum period of follow‐up for studies included in the quantitative synthesis was 1 year.

Overall, little information was reported about how peer leaders (PLs) were selected or nominated and/or the extent of training received (see Supporting information, Table S4). In eight studies 19, 20, 33, 34, 35, 37, 39, 52, peers of a similar age were nominated by their classmates, while in four studies, older‐age peers were selected by staff or researchers 32, 36, 51, 53. In other programmes, PLs either volunteered 31, 36, 38 or the age or method of selection was unclear. Few of the interventions were solely peer‐led; in many cases, peers provided part of the curriculum or led discussions or group work, while teachers acted as facilitators or supervisors.

The majority of studies (*n* = 14) were judged to be of unclear risk of selection bias, as insufficient information was provided around the methods of random sequence generation and/or allocation concealment (Supporting information, Table S5). Most (*n* = 14) were also at high risk of performance bias owing to the difficulty of blinding, although this is generally not possible in interventions of this nature. In many cases, insufficient information and/or poor reporting made it difficult to judge risk of bias.

## Tobacco use

**Figure 2:**
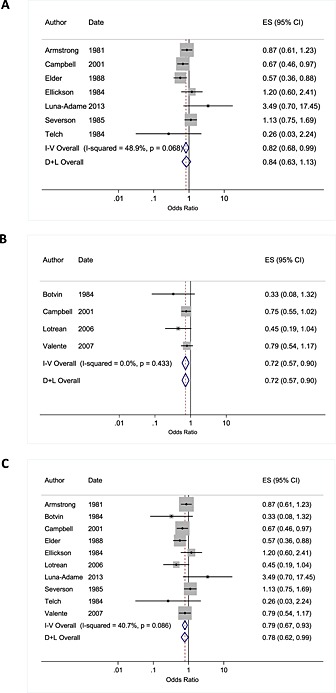
Meta‐analysis of studies showing the impact of peer interventions in relation to weekly or monthly smoking in young people among studies reporting unadjusted data (a); adjusted data (b) and all pooled studies (c). Pooled effect estimates are shown for fixed and random effects models. Date reflects start date of study. or where such data were not provided, the date of the first paper

First, we pooled data for seven studies that reported unadjusted data, representing 12 228 young people in 182 schools. Meta‐analysis demonstrated that there was limited evidence that those receiving a peer‐led intervention had lower odds of weekly or less frequent smoking compared to those in the control arm (OR = 0.84, 0.63–1.13, *P* = 0.253; Fig. 2) and we identified heterogeneity between studies (*I* ^2^ = 49%, χ^2^ = 11.73, *P* = 0.068). The fixed‐effect model gave stronger evidence of effect (OR = 0.84, 0.63–1.13, *P* = 0.037).

Secondly, pooled analysis of adjusted estimates from four studies provided a sample of 10 767 young people in 97 schools. We found moderate evidence that the odds of weekly or monthly smoking were lower among those in the peer‐led arm compared to control (OR = 0.72, 0.57–0.90, *P* = 0.005; *I* ^2^ = 0%, χ^2^ = 2.74, *P* = 0.433; Fig. 2). The fixed‐effect model gave the same result.

Importantly, pooling of all data from adjusted and unadjusted estimates (providing a sample of 13 706 young people from 220 schools) showed that those receiving a peer‐led intervention had lower odds of weekly or monthly smoking compared to those in the control arm (OR = 0.78 0.62–0.99, *P* = 0.040; *I* ^2^ = 41%, χ^2^ = 15.17, *P* = 0.086; Fig. 2) and again, the effect estimate from the fixed‐effect model was similar (Fig. 2).

Sensitivity analyses were conducted to examine the effect of excluding: studies for which assumptions were made for data analysis 30; studies which reported the outcome variable at lower frequency (i.e. in the past 12 months rather than weekly or monthly) 35; and the effect of including a study which compared peer‐led intervention to teacher‐led intervention 33. In all cases, syntheses gave similar estimates of effect. Findings did not change markedly when different values of the ICC were used to account for clustering (Supporting information, Table S6).

We were unable to investigate reasons for heterogeneity owing to the small number of studies. The funnel plot was suggestive of asymmetry with few small studies reporting negative effects, which may reflect publication bias or poor methodological design, and which could potentially have led to an inflated estimate of effect (Egger test: *n* = 10 studies; Egger test coefficient − 1.56, *P* = 0.08). However, as this test has low statistical power when there are few studies, this result should be interpreted with caution 55.

## Alcohol use

**Figure 3:**
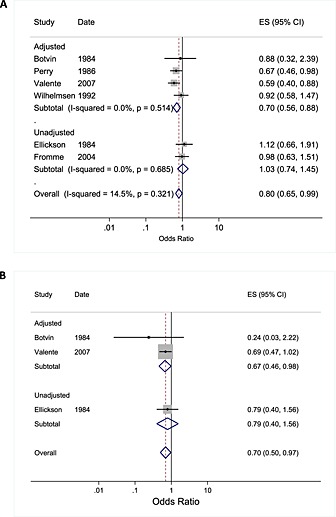
Meta‐analysis of peer‐led studies targeting alcohol use (a) and cannabis use (b) in young people grouped by adjustment of outcome data. Date reflects start date of study, or where such data were not provided, the date of the first paper from the study. For meta‐analysis (b), fixed and random effect models gave the same overall estimate of effect

Fewer studies targeted alcohol use (*n* = 6 studies), thus the total sample size was smaller than that relating to tobacco use, including 1699 young people in 66 schools and one university. Four of these studies were conducted in the United States. Pooled analysis of all studies showed weak evidence of lower odds of alcohol use among those in the peer‐led arm compared to control (OR = 0.80, 95% CI = 0.65–0.99, *P* = 0.036; *I* ^2^ = 14.5%, χ^2^ = 5.85, *P* = 0.321; Fig. 3). The fixed‐effect model produced a similar result (OR = 0.79, 95% CI = 0.66–0.96, *P* = 0.017). Sensitivity analysis, which excluded one study for which assumptions were made to facilitate analysis 51 altered the effect estimate, however (OR = 0.85, 0.66–1.08, *P* = 0.191; *I* ^2^ = 7.4%, χ^2^ = 4.84, *P* = 0.304), while meta‐analysis of unadjusted estimates also gave a null result (*n* = 597 young people in 20 schools; Fig. 3). Synthesis of adjusted estimates only (a sample including 1102 young people in 46 schools) showed stronger evidence of effect (OR = 0.71, 95% CI = 0.56–0.89, *P* = 0.003; Fig. 3).

## Cannabis use

Only three studies targeted cannabis use, all of which were school‐based studies conducted in the United States (see Table 1). Pooling of these studies, which represented 976 young people in 38 schools, demonstrated that the odds of cannabis use were lower for those in the peer‐led condition compared to control (OR = 0.70, 0.50–0.97, *P* = 0.034; *I* ^*2*^ = 0.0%, χ^2^ = 1.0, *P* = 0.605; Fig. 3).

## Adverse effects of peer‐led interventions

While effect estimates from meta‐analyses favoured the intervention, two studies highlighted that the peer‐led intervention may, in fact, enhance tobacco or alcohol use among certain higher‐risk groups. One of these studies 20 involved identification of PLs via social network nominations, and the use of teams alongside small group discussion and role‐play with the peer leader. Data demonstrated that substance use was higher among participants with existing networks of substance‐using peers (b = 0.17, 0.08–0.26, *P* < 0.01), while substance use was reduced mainly for those students who nominated students who reported low levels of substance use. A second study 30 demonstrated that a greater proportion of baseline smokers in the peer‐led arm reported smoking in the past month, monthly and weekly smoking (63.2 versus 48.9%; 54 versus 43.3%; and 34.6 versus 26.4%, respectively). Among these individuals, pro‐smoking attitudes were more prevalent and a markedly higher proportion were around peers who smoked and had a best friend who reported smoking.

## Discussion

We have quantified for the first time, to our knowledge, evidence of the impact of peer‐based interventions on substance use among young people. Our findings suggest that peer interventions have a role to play in preventing tobacco, alcohol and possibly also cannabis use during adolescence. The pooling of nine studies incorporating over 13 700 young people in 220 schools suggested that weekly or less frequent smoking was lower among those who received a peer‐led intervention compared to control (OR = 0.78 0.62–0.99, *P* = 0.040), while pooling of six studies provided weak evidence supporting an association between peer‐led interventions and lower odds of alcohol use (OR = 0.80, 95% CI = 0.65–0.99, *P* = 0.036). Meta‐analysis of three studies including 976 young people in 38 schools also suggested that peer‐led interventions reduced cannabis use (OR = 0.70, 95% CI = 0.50–0.97, *P* = 0.034).

Our findings corroborate and strengthen earlier reviews, which suggested that the inclusion of peers in public health interventions would have benefits in preventing harmful behaviours during adolescence 17, 18, 28, 56. Our findings also suggest that there may be scope to consider the more extensive trial and implementation of peer‐led intervention models, given current gaps in the evidence base regarding the prevention of substance use among young people. The majority of studies targeting tobacco use were conducted in school settings where peer leaders contributed by delivering part or all of the curriculum, suggesting that interventions in this particular setting may be appropriate. Notably, cannabis was the only illicit drug targeted by the studies identified.

## Strengths and weaknesses

The strengths of our study include the thorough literature search across multiple databases, the focus on three different substances and the quantification of effect through pooled analyses, which has not been conducted in this field to date. However, there are several limitations to our review and the evidence presented.

First, when searching the grey literature we prioritized large national and international organizations known for their work in the field rather than comprehensively searching the websites of all organizations working in this field, so there is a small risk that we failed to include all relevant studies. Secondly, all included studies were subject to bias, and the quality of evidence for each outcome [under, for example, a classification system such as GRADE (Grading of Recommendations Assessment, Development and Evaluation 57)] would be considered to be low, owing primarily to the poor quality of data reporting in the included studies. In many cases, methods of randomization and allocation concealment were not provided, the extent of blinding was unclear and attrition was relatively high in some studies. The majority of school‐based studies failed to adjust adequately for clustering, although we accounted for this for during analysis, and we were required to manipulate the data in order to complete quantitative synthesis.

Thirdly, there were insufficient data to compare the impact of interventions in different risk groups (e.g. among experimental substance users) or by gender, ethnicity or socio‐economic status, and it is possible that effectiveness differed in particular groups. We also excluded studies assessing the impact of brief interventions and those targeted to individuals with a clinical disorder. Our choice to analyse data taken at the end of intervention, at which point the intervention effect may be greatest, may have led to an overestimation of effect, although we may also have missed longer‐term beneficial impacts. Lastly, the majority of studies were conducted in the United States, with 10 of 17 studies (59%) trialled in the 1980s and 1990s, during which time cultural norms and the prevalence of behaviours would have been different. Thus, the generalizability of our findings may be limited, given this geographical and cultural context. Nonetheless, consistent evidence was generated with implications for practice and future research.

## Implications and other evidence

Our systematic review and meta‐analysis supports previous evidence 18, 27, 28 by highlighting the promise and effectiveness of peer‐led interventions. However, our study goes further in strengthening the evidence by including more recent studies and by providing a quantified estimate of the effect of such interventions in relation to tobacco, alcohol and cannabis use.

The intervention effects observed may be explained, in part, by the fact that peers are likely to be embedded in social groups and communities, may share social status and cultural background and may have greater credibility than adults or professionals, such that behaviour change messages may resonate to a greater extent with young people. There may also be benefits to the peer leaders themselves through enhanced confidence, self‐esteem, communication skills and behaviour change 17 although this was not examined in the eligible studies in our review.

The evidence suggests that peer‐based interventions may be effective in relation to tobacco, alcohol and cannabis use among young adults compared to controls, and that a variety of different approaches may be effective. Among those interventions that were the most effective 19, 20, 31, 54, however, there was no clear pattern of factors associated with impact, such as shared intervention domains, duration of intervention or underlying theory. In addition, no clear message emerged regarding whether peers, teachers or professionals were more effective at instigating behaviour change. One promising approach 19, which has been implemented in the South West of England and south Wales, utilized successfully the diffusion of innovations model 58. This model explains how innovations are communicated to members of a social system through various channels over time 58. In this programme (ASSIST), ‘peer supporters’ communicated smoking prevention messages informally to their friends as part of their usual social interactions. The same model has effectively underpinned other studies targeting sexual risk behaviour 26, the use of contraception 59, cardiovascular disease prevention 60 and drug use 61, suggesting that there may be scope for further investigation of the use and effectiveness of this approach, particularly where it involves the activation and harnessing of peer networks.

A second programme 31 implemented an intervention over a number of school years, culminating in one‐to‐one communication with participants. Taken together with the finding that interventions that included a booster session were more effective than those without 30, 31, 32, 51, the data suggest that the repeated communication of health messages over an extended time‐period may have a more pronounced impact on the behaviour of young people.

Of concern, however, was the finding that in two interventions, young people reported greater engagement in substance use following receipt of the peer‐led programme 20, 62. This phenomenon has been reported elsewhere for peer‐led and multi‐component youth programmes 63, 64, particularly among high‐risk groups 63. Similarly, in the two studies in our review, the effect was attributed to involvement with existing networks of substance‐using peers in one study 20, while in the other it was noted that increased rates of smoking were observed among those with pro‐smoking attitudes and a substantial proportion of tobacco‐using friends 62. In order to heed the messages of the intervention, young people in these groups would therefore have needed to reject the norms of their peer group and thus risk social isolation. These findings suggest that interventions (including peer‐based models) need to take account of peer norms and peer influences in young people's friendship groups and social networks, while preventive messages may need to be targeted appropriately to different risk groups to maximize effectiveness and to shift norms for all young people across the spectrum of substance use.

## Conclusions

We have identified evidence that peer‐led interventions can be effective in preventing tobacco, alcohol and possibly cannabis use among young people, providing scope for considering the further development and evaluation of such programmes to strengthen the evidence base around effective means of prevention. Our findings, however, are somewhat limited by the poor quality of the evidence. In support of others, we have identified a need for robust, rigorously conducted studies that have longer follow‐up duration, are conducted in a range of geographical contexts, and which assess impacts in different risk and socio‐demographic groups. Further research relating to peer‐based interventions should also include process evaluations, to assess issues around implementation, receipt, fidelity, reach and the setting of an intervention, and to ascertain the views and experiences of the peer leaders themselves.

### Declarations of interests

R.C. is a Director of Decipher Impact, a not‐for‐profit spin‐out company owned wholly by the Universities of Cardiff and Bristol, which licenses and supports the delivery of evidence‐based public health interventions. R.C. authored the included publication, Campbell *et al*. 19. The intracluster correlation coefficient from this study was used to adjust data reported by other studies, where necessary.

## Supporting information

Supporting info item
