# Supplementary material for: Peer‐led interventions to prevent tobacco, alcohol and/or drug use among young people aged 11–21 years: a systematic review and meta‐analysis
Source: Addiction. 2016 Feb 9;111(3):391–407. doi: 10.1111/add.13224 (PMC4833174; doi:10.1111/add.13224)
Supplement: Supplementary file 1 — Supporting info item [file ADD-111-391-s001.docx]

**Supplementary Material**

**Table S1. Search strategy (Medline)**

| 1 | adolescent/ or child/ |
| --- | --- |
| 2 | (school* or student* or child* or pupil*).tw. |
| 3 | (Adolescen* or teen* or young person or young people or youth* or young adult* or early adult* or juvenile* or minor? or emerging adult* or girl* or boy* or young m#n or young wom#n or young male* or young female* or under 18* or sixth-form* or secondary education or tertiary education or higher education or further education).mp. |
| 4 | or/1-3 |
| 5 | exp peer-group/ |
| 6 | (peer* adj3 (educat* or promot* or intervention* or program* or train* or counsel* or advis* or lead* or tutor* or advocat* or teach* or taught* or help* or instruct* or manag* or assist* or led or deliver* or directed* or involve* or participat* or support* or adviser* or advisor* or approach*)).mp. |
| 7 | (teen* adviser* or teen* advisor* or teen* tutor* or teen* trainer* or teen* instructor* or teen* leader* or teen* led or teen* delivered or teen* directed or teen* planned or teen* promoted or teen* taught).tw. |
| 8 | (adolescent* adviser* or adolescent* advisor* or adolescent* tutor* or adolescent* trainer* or adolescent* instructor or adolescent* leader* or adolescent* led or adolescent* delivered or adolescent* directed or adolescent* planned or adolescent* promoted or adolescent* taught).tw. |
| 9 | (pupil* adviser* or pupil* advisor* or pupil* tutor* or pupil* trainer* or pupil* instructor or pupil* led or pupil* delivered or pupil* directed or pupil* planned or pupil* promoted or pupil* taught).tw. |
| 10 | (student* adviser* or student* advisor* or student* tutor* or student* trainer* or student* instructor* or student* leader* or student* led or student* delivered or student* directed or student* planned or student* promoted or student* taught).tw. |
| 11 | (young people adviser* or young people advisor* or young people led or young people delivered or young people directed or young people planned or young people promoted or young people taught).tw. |
| 12 | or/5-11 |
| 13 | exp Drinking Behavior/ |
| 14 | exp Alcohol-Related Disorders/ |
| 15 | ((alcohol* or ethanol or beer or cider or wine or spirit* or alcopop*) adj3 (use* or usage* or using or intake or consum* or drink* or misus* or abus*)).mp. |
| 16 | ((alcohol* or drink* or ethanol) adj3 (excess* or binge* or binging or intoxicat* or poison* or risk* or depend*)).mp. |
| 17 | or/13-16 |
| 18 | exp "tobacco use disorder"/ |
| 19 | smoking/ |
| 20 | smoking.mp. |
| 21 | ((tobacco or cigarette* or nicotine) adj3 (addict* or use* or usage or using or intake or consum*)).mp. |
| 22 | or/18-21 |
| 23 | cannabis/ or exp street drugs/ or marijuana smoking/ or Drug-Seeking Behavior/ or Substance-Related Disorders/ or substance abuse, intravenous/ |
| 24 | ((marijuana or cannabis or recreational drug* or class c or substance) adj3 (abus* or use* or using or usage or misus* or smok* or addict* or depend*)).mp. |
| 25 | ((Class a or class b or drug* or cocaine or ecstasy or mdma or glue or gas or aerosol* or solvent* or inhal?nt* or magic mushroom* or crack or ketamine or heroin or morphine or narcotic* or opiat* or opioid* or popper* or lsd or methamphetamine* or amphetamine*) adj3 (abus* or addict* or depend* or inhal* or misus* or sniff* or use* or using or usage)).mp. |
| 26 | or/23-25 |
| 27 | (randomized controlled trial or controlled clinical trial).pt. |
| 28 | (randomi#ed or placebo or randomly).ab. |
| 29 | trial.ti. |
| 30 | clinical trials as topic.sh. |
| 31 | or/27-30 |
| 32 | exp animals/ not humans.sh. |
| 33 | 31 not 32 |
| 34 | 4 and 12 and (17 or 22 or 26) |
| **35** | **34 and 33** |

**Material S2**

**Methodology: Additional information regarding data manipulation and adjustment for clustered data**

In a minority of included studies,[1-3] data regarding the specific numbers of participants in different study arms were not available. In these studies, it was assumed that participants were evenly distributed between study arms and sensitivity analyses were conducted to assess the impact of including or excluding these studies in meta-analyses. For one study,[4] in which data were presented according to baseline smoking status, the proportion of baseline non-smokers and experimental smokers were obtained from an article regarding the same study published at an earlier stage of follow up.[5] In one other study in which standard deviations were not reported,[6] we used standard deviations from a paper published on the same intervention trialled in different schools in the same state and country.[7]

A number of studies did not account for clustering despite being school-level interventions. For these studies, we adjusted for clustering as outlined in the Cochrane Handbook (section 16.3.4-5)[8] using the intracluster correlation coefficient (ICC) reported by Campbell et al.,[9]. We used the upper and lower boundaries of the 95% confidence interval (CI) for the ICC in sensitivity analyses (with the exception of one study,[10] for which we used an upper boundary consistent with an ICC upper level reported in the paper).

*See page 13 for references.*

**Table S3. Duration of intervention and follow-up for studies included in the quantitative synthesis**

| **Study first author, date** | **Duration of follow-up**  **(after baseline)^a^** | **Duration of intervention** | **Number of intervention sessions** |
| --- | --- | --- | --- |
| Albrecht, 2006^b^ | 8 weeks | 8 weeks | 8 sessions |
| Armstrong, 1990 | 18 months | 6 months | 5 sessions |
| Bobrowski, 2014 | 27 months | 12 months^c^ | 5 sessions (4^th^ grade)  6 sessions (5^th^ grade) |
| Botvin, 1990 | 16 months | 18 months^d^ | 20 sessions (7th grade)  10 sessions (8th grade) |
| Campbell, 2008 | 3-4 months | 10 weeks | Informal conversations |
| Elder, 1993 | 30-32 months^d^ | 30-32 months^d^ | 10 sessions (7th grade)  8 sessions (8th grade)  telephone and mail program (9th grade) |
| Ellickson, 1990 | 15 months | 24 months | 8 sessions (7th grade)  3 sessions (8th grade) |
| Fromme, 2004 | 6 months | 4 hours | 2 sessions |
| Lotrean, 2010 | 10 months | 5 weeks | 5 sessions |
| Luna-Adame, 2013 | 32 months | 18 months | 24 sessions (8^th^ grade)  12 sessions (9^th^ grade) |
| Murray, 1987 | 18-20 months | 6 months | 5 sessions |
| Perry, 1989 | 3 months | 8 weeks | 5 sessions |
| Rosenblum, 2008 | 12 months | 12 months | Information not provided |
| Severson, 1991 | 12 months | 2-3 weeks | 7 sessions |
| Telch, 1990 | 6 months | 3 weeks | 5 sessions |
| Valente, 2007 | 12 months | 3-4 weeks | 12 sessions |
| Wilhelmsen, 1994 | 3 months | 2 months | 10 sessions |

^a^ For studies included in quantitative syntheses, at time point from which data extracted.

^b^ Period of follow-up from baseline not provided; data illustrate follow up from randomization

^c^ Note that the intervention duration in each school year was 12-15 weeks but since the program ran over consecutive school years, the total duration is considered to be 12 months. The intervention duration is also calculated this way for the additional interventions which took place over consecutive school years.

^d^ Approximate value based on information presented in paper

**Table S4. The role, selection and training of peer leaders (PLs) involved in the included interventions**

| **Author** | **Role of peer leaders** | **Selection of peer leaders** | **Training of peer leaders** |
| --- | --- | --- | --- |
| Albrecht 2006;  Albrecht 1998 | - ‘Buddies’ reinforced smoking cessation strategies and provided social support to the participant. Buddies attended all program sessions to provide peer support. | - A ‘buddy’, who was a non-smoking female of similar age, was chosen by the participant. | - Not stated. |
| Armstrong 1990;  Shean 1994 | - PLs led the programme classes whilst teachers acted as facilitators | - Same-age PLs were selected by their classmates. | - PLs received training but details are not provided. |
| Bobrowski 2014 | - PLs lead the sessions with teachers, facilitate small group discussions and introduce activities, games and role-playing. | - PLs are selected but methods of selection are not provided. | - PLs were trained by teachers. |
| Botvin 1990;  Botvin 1984 | - PLs led the intervention booster sessions | - PLs were individuals in 10^th^, 11^th^ and 12^th^ grade. - PLs were recruited by school bulletins, posters, and teachers. - They were selected on the basis of popularity, responsibility, self-confidence, interest in the program, and willingness to make the necessary commitment. Those judged to be good role models, with high credibility among students and with appropriate attitudes were selected. PLs were interviewed by a member of the project staff and a teacher. | - One four-hour workshop conducted by research project staff members, which involved discussion, demonstration, activities and training in classroom management skills. - Content included: the rationale for the intervention, an overview of the curriculum, implementation guidelines, and demonstration of selected classroom exercises. - Additional briefing sessions included more specific information, and a curriculum containing detailed lesson plans was provided. |
| Campbell 2008 | - ‘Peer supporters’ undertook informal conversations with peers about smoking when opportunities arose. | - Influential ‘peer supporters’ were nominated by students in the same year group. Students were asked who they respected, who were good leaders in sports or other group activities, and who they looked up to. - Peer supporters who smoked could be involved if they committed to trying to stop smoking. | - Two-day training event facilitated by external trainers and health promotion specialists. - Content included information about: risks of smoking, benefits of remaining smoke-free, communication and listening skills, group work, team building, ways of giving and receiving information, cooperation and negotiation, conflict resolution, assertiveness, sensitivity to others, attitudes to risk taking, decision-making, and enhancement of their confidence and self-esteem. - Role play, research, group work, discussion and games were included. |
| Elder 1993; Eckhardt 1997 | - PLs led the intervention sessions. | - Undergraduate volunteer group leaders from San Diego State University. They received units of college credit for their involvement. | - Approximately 15 hours of training from the research staff in teaching effectively and how to implement the lessons. - Proficiency was evaluated and feedback was given during training and in the course of the intervention. |
| Ellickson and Bell 1990;  Ellickson and Bell 1990^b^ | - PLs assisted teachers in delivering programme content for all sessions. They provided personal examples of effective resistance and helped students to believe that they could resist substance use. | - Older teen leaders from neighbouring schools who assisted the teachers in delivering the curriculum. - PLs were expected to be role models for successful non-use and to provide personal examples of effective resistance to substance use. | - Not stated |
| Fromme and Corbin 2004 | - PLs facilitated the group sessions. | - Students who volunteered to take on the role. | - Training in motivational interviewing techniques and strategies for facilitating group discussions in a non-judgemental and empathic manner. |
| Lotrean 2010 | - PLs served as chairmen of small activity groups, which involved summarising activities, simulating group work and presenting outcomes of group activities. Teachers co-ordinated lessons and assisted the peer leaders. | - Students from the same class. No detail is provided about the selection of PLs. | - Provision of a manual providing guidance on the content of group discussions, and a one-hour information session, which provided information about the content and the characteristics of the programme. |
| Luna-Adame 2013 | - PLs implemented the intervention program. | - University psychology students who volunteered to take on the role. | - PLs auditioned in a role play and the best underwent a 9-month training course with weekly 90-minute sessions. At 15 days before the 1^st^ year of the program and 15 days before the 2^nd^ year of the program, they participated in an intensive 7-day course of 7 hours per day. |
| Murray 1988;  Murray 1987 | - PLs led the classroom sessions while the teachers acted as facilitators | - Same age peer leaders selected by classmates based on who they admired and respected. The three individuals receiving the most votes were selected. | - Two training sessions conducted by project staff. - Content covered: group dynamics, leadership skills, and rehearsal of the activities in the intervention. - An outline of each day’s activities was also provided. |
| Perry 1989; Perry and Grant 1991 | - PLs directed approximately 70% of the content of the classroom sessions | - Peers who volunteered (Chile, Norway) or were selected by teachers (Australia, Swaziland). | - 4-12 hours of training conducted by the researchers. - Content covered: skills in presenting information, organising small group discussions, leading role plays and synthesizing the information learned. - Manuals with a script for the sessions were also provided. |
| Rosenblum 2005 | - Mentoring and attendance at support groups with the mentee. | - Peer mentors (13-19yrs) identified from a list of current clients of the community organisation. 90% had completed the mentoring programme themselves. | - Training over a three month period. Extended training was received from adult mentors. This included ongoing supervision and participation in a 15-session training curriculum covering issues such as relationships, HIV, sex, substance use, and stress management. |
| Severson 1991 | - PLs led activities in five of seven classroom sessions. | - PLs were nominated by students as people they liked and admired. | - Training was conducted on two occasions by project staff. - Content included review of each activity, watching excerpts of the videotapes and practicing and role-playing associated activities. |
| Telch 1990 | - PLs contributed to sessions that included videotape training by providing role models advocating a non-smoking position | - Same age PLs nominated by classmates. Students were asked to write down the names of two boys and two girls who they respected and who would be good at leading classroom discussions on why students should not smoke. - Those receiving the most votes were selected. | - One hour training session conducted by a member of the research team providing information about smoking and ‘group processes’. Training included an opportunity to rehearse the leading of discussion groups. |
| Valente 2007 | - PLs facilitated group discussion and led role-played assertive communication strategies. | - PLs were nominated by their classmates on the basis of who they thought would make good leaders. Those who received the most nominations were selected. | - Training of approximately one hour in how to facilitate discussion and manage group interaction. - Training covered normative restructuring about drug use and PLs were encouraged to embrace anti-substance use norms. |
| Wilhelmsen 1994 | - PLs acted in concert with teachers to implement pre-planned classroom activities (highly role specified version, HRS) or co-operated with teachers to take responsibility for implementing the programme (less role specified version, LRS). | - PLs were nominated by students on the basis of who they thought would do a good job as leaders. An equal number of male and female PLs were chosen, 4 per class for the HRS version and 2 per class for the LRS version. | - A programme manual was provided for teachers and PLs, but no details around specific training for PLs are provided. |

**Table S5. Risk of bias in included studies**

| **Study** | **Sequence generation** | **Allocation concealment** | **Blinding (participants)** | **Blinding (outcome assessment)** | **Incomplete outcome data** | **Selective reporting** | **Other** |
| --- | --- | --- | --- | --- | --- | --- | --- |
| Albrecht, 2006 | Low | Unclear | High | Unclear | High | Unclear | Low |
| Armstrong, 1991 | Unclear | Unclear | Low | Low | Unclear | Unclear | High |
| Bobrowski, 2014 | Unclear | Unclear | High | Unclear | Low | Unclear | Unclear |
| Botvin, 2001 | Unclear | Unclear | Unclear | Unclear | Unclear | High | High |
| Campbell, 2008 | Low | Low | Low | Unclear | Low | Low | Low |
| Elder, 1993 | Unclear | Unclear | High | Unclear | Low | Unclear | Low |
| Ellickson, 1990 | Unclear | Unclear | High | Unclear | Low | Unclear | Low |
| Fromme, 2004 | Unclear | Unclear | High | Unclear | Unclear | Unclear | Low |
| Lotrean, 2010 | Low | Unclear | High | Unclear | Low | Unclear | Low |
| Luna-Adame, 2013 | Unclear | Unclear | High | Unclear | Unclear | Low | Low |
| Murray, 1987 | Unclear | Unclear | High | Unclear | High | Unclear | Unclear |
| Perry, 1989 | Unclear | Unclear | High | Unclear | Low | Unclear | Low |
| Rosenblum, 2008 | Unclear | Unclear | High | Unclear | Unclear | High | High |
| Severson, 1991 | Unclear | Unclear | High | Unclear | High | Unclear | Low |
| Telch, 1990 | Unclear | Unclear | High | Unclear | Unclear | High | Unclear |
| Valente, 2007 | Unclear | Unclear | High | High | Unclear | Unclear | Low |
| Wilhelmsen, 1994 | Unclear | Unclear | High | Unclear | Unclear | Low | Low |

**Table S6. Sample sizes and effect estimates from sensitivity analyses conducted around the intra-cluster correlation coefficient (ICC) used in the main meta-analyses.**

**A. Tobacco use**

| **ICC** | **Adjustment** | **Studies (n)** | **Sample size** | **Odds Ratio** | **p value** |
| --- | --- | --- | --- | --- | --- |
| ICC provided^a^ | Unadjusted | 6 | 12,228, 182 schools | 0.84, 0.63-1.13 | 0.253 |
|  | Adjusted | 4 | 10,767, 97 schools | 0.72, 0.57-0.90 | 0.005 |
|  | All | 10 | 13,706, 220 schools | 0.78, 0.62-0.99 | 0.040 |
| Lower level^b^ | Unadjusted | 6 | 14,095, 182 schools | 0.86, 0.64-1.17 | 0.339 |
|  | Adjusted | 4 | 10,638, 97 schools | 0.64, 0.46-0.89 | 0.009 |
|  | All | 10 | 15,384, 220 schools | 0.76, 0.59-0.99 | 0.045 |
| Upper level^c^ | Unadjusted | 6 | 11,758, 182 schools | 0.84, 0.64-1.09 | 0.189 |
|  | Adjusted | 4 | 10,104, 97 schools | 0.73, 0.58-0.92 | 0.008 |
|  | All | 10 | 12,513, 220 schools | 0.79, 0.64-0.98 | 0.030 |

**B. Alcohol use**

| **ICC^a^** | **Adjustment** | **Studies (n)** | **Sample size** | **Odds Ratio** | **p value** |
| --- | --- | --- | --- | --- | --- |
| ICC provided | Unadjusted | 2 | 597, 20 schools | 1.03, 0.74-1.45 | 0.848 |
|  | Adjusted | 4 | 1,279, 46 schools | 0.71, 0.56-0.89 | 0.003 |
|  | All | 6 | 1,876, 66 schools | 0.80, 0.65-0.99 | 0.036 |
| Lower level | Unadjusted | 2 | 752, 20 schools | 1.05, 0.77-1.43 | 0.752 |
|  | Adjusted | 4 | 1898, 46 schools | 0.73, 0.60-0.89 | 0.002 |
|  | All | 6 | 2650, 66 schools | 0.82, 0.67-1.00 | 0.051 |
| Upper level | Unadjusted | 2 | 540, 20 schools | 1.03, 0.73-1.47 | 0.857 |
|  | Adjusted | 4 | 1,023, 46 schools | 0.69, 0.54-0.89 | 0.004 |
|  | All | 6 | 1,563, 66 schools | 0.80, 0.64-0.98 | 0.035 |

**C. Cannabis use**

| **ICC^a^** | **Adjustment** | **Studies (n)** | **Sample size** | **Odds Ratio** | **p value** |
| --- | --- | --- | --- | --- | --- |
| ICC provided | N/A | 3 | 1,158, 38 schools | 0.70, 0.50-0.97 | 0.034 |
| Lower level | N/A | 3 | 1,798, 38 schools | 0.71, 0.53-0.95 | 0.022 |
| Upper level | N/A | 3 | 1,004, 38 schools | 0.70, 0.50-0.99 | 0.043 |

^a^ ICC of 0.03 provided in Campbell R et al. An informal school-based peer-led intervention for smoking prevention in adolescence (ASSIST): a cluster-randomized trial. Lancet (2008); 371: 1595-1602.

^b^ ICC equivalent to the lower boundary of the 95% confidence interval reported by Campbell et al (2008).

^c^ ICC equivalent to the upper boundary of the 95% confidence interval reported by Campbell et al (2008).

N/A: not applicable.

**References (relevant to Material S2)**

1. Ellickson, P.L., Bell, R.M., *Drug Prevention in Junior High: A Multi-Site Longitudinal Test.* Science, 1990. **247**(4948): p. 1299-1305.

2. Botvin, G.J., et al., *A cognitive-behavioral approach to substance abuse prevention: One-year follow-up.* Addictive Behaviors, 1990. **15**(1): p. 47-63.

3. Perry, C.L., et al., *WHO collaborative study of alcohol education and young people: Outcomes of a four-country pilot study.* International Journal of the Addictions, 1989. **24**(12): p. 1145-1171.

4. Murray, D.M., et al., *The prevention of cigarette smoking in children: two- and three-year follow-up comparisons of four prevention strategies.* Journal of Behavioral Medicine, 1987. **10**(6): p. 595-611.

5. Arkin, R.M., Roemhild, H.F., Johnson, C.A., Luepker, R.V., Murray, D.M., *The Minnesota smoking prevention program: A seventh-grade health curriculum supplement.* The Journal of School Health, 1981. **51**: p. 611-616.

6. Valente, T.W., et al., *Peer acceleration: effects of a social network tailored substance abuse prevention program among high-risk adolescents.* Addiction (Abingdon, England), 2007. **102**: p. 1804-15.

7. Sussman, S., et al., *One-year outcomes of a drug abuse prevention program for older teens and emerging adults: Evaluating a motivational interviewing booster component.* Health Psychology, 2012. **31**(4): p. 476-485.

8. Higgins J., P., T, Green, S., *Cochrane Handbook for Systematic Reviews of Interventions*. 2008, Chichester: John Wiley and Sons.

9. Campbell, R., et al., *An informal school-based peer-led intervention for smoking prevention in adolescence (ASSIST): a cluster randomised trial.* Lancet, 2008. **371**(9624): p. 1595-602.

10. Lotrean, L.M., et al., *Evaluation of a peer-led smoking prevention programme for Romanian adolescents.* Health Education Research, 2010. **25**(5): p. 803-14.
